# Co-circulation of dengue, chikungunya, and Zika viruses and cross-protection

**DOI:** 10.26633/RPSP.2019.75

**Published:** 2019-08-30

**Authors:** Beuy Joob, Viroj Wiwanitkit

**Affiliations:** 1 Sanitation 1 Medical Academic Center Sanitation 1 Medical Academic Center Bangkok Thailand Sanitation 1 Medical Academic Center, Bangkok, Thailand; 2 Department of Community Medicine Department of Community Medicine Dr DY Patil University Pune India Department of Community Medicine, Dr DY Patil University, Pune, India

To the Editor:

We read the publication on “Co-circulation of dengue, chikungunya, and Zika viruses in Colombia from 2008 to 2018” with a great interest (1). Rico-Mendoza et al. concluded that “The decrease in the number of dengue cases after co-circulation of the three viruses could indicate possible cross-protection” (1). We would like to share ideas on this report. An interesting question is whether there is a cross-protection or not. Based on our setting in Indochina, a similar co-circulation of the three viruses presently exists but there has never been any decrease in number of dengue. In addition, there is no clear report showing that there is a cross-protection due to the three virus co-circulation. Priyamvada et al. mentioned that there might be a common host immune response among viruses due to viral structural similarity, but it is still no data regarding the immunoprotection (2). Based on a recent publication by Wen et al., it is observed that there might be a cross protection via CD8+ but Wen et al. mentioned that “passive transfer studies suggest that DENV-immune serum does not protect against ZIKV infection” (3). Based on the data by Rico-Mendoza et al., there is also a fluctuation of the number of the cases (such as between 2016 and 2018) (1). The fluctuation, increased and decreased rate, might be due to several factors including to the environmental factors that might stimulate or suppress the epidemic of the vector borne disease. Due to the worldwide concern on Zika virus, there might be some intensified prevention program against mosquito borne disease.

## Disclaimer.

The author holds sole responsibility for the views expressed in the manuscript, which may not necessarily reflect the opinion or policy of the RPSP/PAJPH and/or PAHO.

A reply to this letter is avaliable at: https://doi.org/10.26633/RPSP.2019.77
